# The Quality and Cultural Safety of Online Osteoarthritis Information for Affected Persons and Health Care Professionals: Content Analysis

**DOI:** 10.2196/57698

**Published:** 2024-10-18

**Authors:** Smita Dhakal, Shermeen Merani, Vandana Ahluwalia, Marisa Battistella, Cornelia M Borkhoff, Glen Stewart Hazlewood, Aisha Lofters, Deborah A Marshall, Crystal MacKay, Anna R Gagliardi

**Affiliations:** 1 University Health Network Toronto, ON Canada; 2 William Osler Health System Brampton, ON Canada; 3 Institute of Health Policy, Management and Evaluation Dalla Lana School of Public Health University of Toronto Toronto, ON Canada; 4 University of Calgary Calgary, AB Canada; 5 Department of Family and Community Medicine University of Toronto Toronto, ON Canada; 6 West Park Healthcare Centre North York, ON Canada

**Keywords:** osteoarthritis, women’s health, equity, educational materials, internet, content analysis, Canada, persons living with osteoarthritis, healthcare professionals, OA care, ethno-culturally women, immigrant women, diverse women, online materials, health information, prevention, management, misinformation, cultural safety, educational materials

## Abstract

**Background:**

Osteoarthritis is more prevalent and severe among women than among men, but women are less likely to access early diagnosis and first-line management, particularly racialized immigrant women. Previous research advocated for greater access to culturally safe osteoarthritis information for both diverse women and health care professionals. The internet can reduce disparities by facilitating access to health information, but online materials can vary in quality.

**Objective:**

This study aimed to assess the quality and cultural safety of online osteoarthritis materials for persons affected by osteoarthritis and health care professionals.

**Methods:**

Content analysis was used to describe publicly available materials on osteoarthritis first-line management developed by Canadian organizations for affected persons or health care professionals. Searching, screening, and data extraction were performed in triplicate. We identified materials by searching Google, MEDLINE, and references of osteoarthritis-relevant guidelines and policies, and consulting our research team and collaborators. We assessed quality using DISCERN (University of Oxford) and a compiled framework for affected persons and health care professionals. We compiled frameworks to assess cultural safety. We derived an overall score, categorized as low (<50%), moderate (50%-69%), or high (≥70%+) for criteria met.

**Results:**

After screening 176 items and eliminating 129, we included 47 osteoarthritis materials published between 2013 and 2023. Of those, 43 were for persons with osteoarthritis, most were developed by charities (n=31, 72.1%), based on expert advice (n=16, 55.2%), and in the format of booklets (n=15, 34.9%) or text on web pages (n=10, 23.3%). Of those, 23.3% (10/43) low, 46.5% (20/43) moderate, and 30.2% (13/43) high scored quality; and 25.6% (11/43), 48.8% (21/43), and 25.6% (11/43) were rated low, moderate, and high cultural safety, respectively. Of the 47 included osteoarthritis materials, 4 were for health care professionals. They were developed by a consortium (2/4, 50%), a charity (1/4, 25%), and a professional society (1/4, 25%), and largely based on expert advice (3/4, 75%). The format included infographics (3/4, 75%) and text on web pages (1/4, 25%). Of those, 25% (1/4), 25% (1/4), and 50% (2/4) were rated low, moderate, and high quality, respectively; and all were rated low for cultural safety. Quality and cultural safety did not appear to be associated with the characteristics of osteoarthritis materials (eg, type of developer, development method, and format).

**Conclusions:**

Overall, included osteoarthritis materials for persons with osteoarthritis and health care professionals were of low to moderate quality and cultural safety. These findings reveal the need for further efforts to improve existing or develop new osteoarthritis materials for both affected persons, including ethnoculturally diverse immigrant women, and health care professionals. Further research is needed to assess the quality and cultural safety of osteoarthritis materials developed by organizations outside of Canada and to establish a framework or instrument to assess cultural safety in the osteoarthritis context.

## Introduction

The internet has become a commonly used way to share health information with patients and health care professionals, alike. For patients, online health information can help people learn about disease prevention or management, prepare them to interact with the formal health care system, supplement advice from health care professionals, connect them to others with a common health concern, link patients with their own health records, or support those without access to care [1]. Associated positive outcomes include increased health care visits, patient question-asking during consultations, adherence to prescribed therapies, and behavioral compliance with self-care [2]. For health care professionals, online health information can help clinicians to answer patient questions, engage in shared decision-making, and support professional development and maintenance of competencies, with a demonstrated positive impact on knowledge and behavior [3,4].

Despite these numerous benefits, the use of the internet to access health information can present challenges to both patients and health care professionals. Inaccurate or incomplete information can misinform users, and poor design or functionality can impede learning, and the problem is widespread [5]. For example, a review of 153 reviews evaluating 11,785 websites offering health information to the public found that most were rated as poor quality [6]. Another review of 282 studies identified many technological barriers to the use of online health information by health care professionals including lack of interactivity, feedback, personalization, cost, and privacy concerns [4]. Therefore, as we strive to enhance widespread access to health information, it is important to assess online sources to identify those of high quality that can be recommended to users.

The internet can be particularly helpful in supporting persons with chronic conditions that benefit from self-management. One such condition is osteoarthritis. Osteoarthritis is an incurable condition affecting millions worldwide that most often is felt in the hips, knees, hands, neck, back, and feet [7]. Osteoarthritis manifests as chronic joint pain, tenderness, swelling, and stiffness, affecting activities of daily living, employment, and quality of life, and leading to other conditions such as heart disease, diabetes, or depression [7,8]. Early intervention is key to prevent worsening and manage symptoms, and involves education about self-management, which can include physical activity, weight loss, and pain management [9,10]. Osteoarthritis is more prevalent and severe among women than among men [11]. However, women are less likely than men to receive self-management advice, particularly racialized immigrant women [12-14]. This may be due to low rates of help-seeking that result from poor mainstream health care experiences [15], and lack of knowledge and skills among clinicians for supporting osteoarthritis self-management among ethnoculturally diverse persons [16,17]. Furthermore, immigrant women have lower rates of physical activity, an important part of osteoarthritis self-management, compared with immigrant men or nonimmigrants and are therefore in need of osteoarthritis self-management advice [18].

A multicountry study showed that access to health information on the internet facilitates health care access and mitigates the negative impact of social and economic determinants of health care access to reduce disparities [19]. In our previous research, ethnoculturally diverse women (White, East Asian, South Asian, and African and Caribbean Black) aged from 40 to 70+ with one or more of hand, hip, knee, and neck osteoarthritis for ages from 5 to 30+ years from across Canada, and women and men health care professionals of various specialties (chiropractor, health care executive, family physician, nurse practitioner, occupational therapist, pharmacist, physiotherapist, and policy maker) of early, mid and later career from across Canada identified and prioritized strategies to improve access to quality of osteoarthritis care for women. Strategies included greater access to publicly available osteoarthritis information for women that accommodates cultural expressions, norms and preferences, and to continuing education for health care professionals on osteoarthritis management and how to tailor osteoarthritis advice to women’s intersectional factors [20,21]. Most research on support for osteoarthritis self-management has focused on the impact of osteoarthritis self-management programs delivered directly by health care professionals [22]. Other research on internet self-management resources has focused on conditions other than osteoarthritis; for example, fatigue among patients with cancer [23,24]. Hence, knowledge is needed on the quality and relevance of online health information for diverse women and health care professionals that could support osteoarthritis self-management.

Given the lack of access to osteoarthritis self-management advice among ethnoculturally diverse women [12-18]; and the important role of online health information in supporting osteoarthritis self-management among such women [19-21], and continuing education among health care professionals [3,4], the aim of this study was to identify online osteoarthritis information to improve osteoarthritis care for ethnoculturally diverse women. More specifically, the objective of this study was to assess the quality and cultural safety of online osteoarthritis information for patients and health care professionals. If identified, we could recommend osteoarthritis materials of high quality and cultural safety to diverse women and health care professionals. If none or few were identified, that would signal the need for de novo development.

## Methods

### Approach

We used content analysis, a methodological approach widely used to analyze the content of written or visual materials [25,26]. More specifically, we applied manifest content analysis, which is a way to describe explicit content without theoretical interpretation. This technique differs from thematic coding typically used to analyze transcripts of interviews or focus groups and involves both quantitative (eg, counting the number of documents that mention a concept) and qualitative (eg, extracting content relevant to a concept) methods. To optimize rigor, we complied with content analysis methods [25,26] and criteria for reporting qualitative research [27]. The research team informed data collection, analysis, and interpretation to further support rigor. The research team was comprised of a 12-member advisory group of diverse women with osteoarthritis; 6 coinvestigators including health care professionals (family physician, rheumatologists, physiotherapist, and pharmacist), and health services researchers with expertise in the topics of osteoarthritis, person-centered care, equity, and women’s health; and 4 collaborators who represented organizations that could apply the findings: 2 advocacy groups for persons with osteoarthritis and 2 health care professional societies. In particular, women advisors, who varied by type of osteoarthritis, age, length of time since osteoarthritis diagnosis, ethnocultural group, and location in Canada, helped us to search for osteoarthritis materials, develop and test the use of analytic frameworks, and summarize the findings.

### Eligibility

Multimedia Appendix 1 provides detailed eligibility criteria. In brief, we included publicly available, online, English-language materials (eg, video, infographic, checklist, booklet, and web page featuring text with or without other materials) that support osteoarthritis first-line management for persons with osteoarthritis or health care professionals who care for such persons; and were developed by Canadian nonprofit organizations (eg, government, academic groups, professional societies, and charities) in 2012 or later, when the Arthritis Alliance Canada issued a framework to improve arthritis prevention and care [28]. These types of organizations are inclusive of multiple sectors, represent a comprehensive list of types of organizations most likely to generate develop and share osteoarthritis materials on the internet, and because they are nonprofit, materials are likely to freely accessible. We included osteoarthritis materials developed or shared by only Canadian organizations for reasons, that (1) this study was 1 part of a multicomponent research study focused on osteoarthritis care in the Canadian setting, (2) the study was funded by the Arthritis Society Canada, and (3) the study aimed to inform policies and practice needed in the Canadian setting to enhance equitable access to quality of osteoarthritis care. Furthermore, restricting to only Canadian osteoarthritis materials enhanced study feasibility; it required considerable time and effort to evaluate the eligible Canadian osteoarthritis materials—we would not have been able to do so for the potentially hundreds or thousands of osteoarthritis materials developed by organizations around the world. We excluded clinical practice guidelines and resources that focused solely on second-line management (eg, joint replacement) given the importance of first-line management in preventing further decline and optimizing the quality of life [9,10].

### Searching and Screening

SM (woman of color, Master of Public Health candidate) and SD (woman of color, Master of Public Health research coordinator) conducted searching and independently screened all items in duplicate between April 12 and May 23, 2023. ARG (woman, PhD-trained principal investigator) provided training and oversight and resolved discrepancies by independent review, and then discussion with SM and SD. SM and SD first conducted searches, capturing the organization, title, and address of potentially relevant items in a Microsoft Excel file; then revisited each item to confirm eligibility. Given no standard method of searching for “gray literature” [29,30], we used a multipronged strategy that included (1) using Google, searched for “osteoarthritis AND Canada OR <each of the provinces>,” and scanned search results on up to 6 pages of 10 results per page, or before relevancy dropped off; (2) consulting members of the aforementioned research team including women advisors, coinvestigators and collaborators plus other professional societies representing health care professionals who care for persons with osteoarthritis (eg, family physicians, physiotherapists, chiropractors, occupational therapists, and pharmacists); (3) scanning the websites of the Arthritis Society, advocacy groups and professional societies; (4) reviewed the content of Canadian guidelines and policies related to osteoarthritis identified in our previous research [31,32]; and (5) Searched MEDLINE for “osteoarthritis AND Canada AND Internet AND health resources OR education, medical, continuing” for articles that reviewed or developed osteoarthritis information.

### Data Extraction

SM extracted data from osteoarthritis materials for persons with osteoarthritis, SD extracted data from osteoarthritis materials for health care professionals, and then each checked the others’ work, consulting with ARG to resolve discrepancies through discussion to achieve 100% agreement. From all materials, we extracted data on osteoarthritis material characteristics (developer name, type of organization, year published, title of osteoarthritis material, web address, development approach, osteoarthritis topic, and objective) and design according to the Workgroup for Intervention Development and Evaluation Research framework (content, format, and delivery) [33].

#### Persons With Osteoarthritis

To assess the quality of osteoarthritis materials for affected persons, we used DISCERN because it is a validated instrument and has been widely used by many others to assess the quality of consumer health information [34]. The 15-item DISCERN instrument assesses reliability (clarity of aims, aims achieved, relevance, transparency about sources of information, date information generated or published, information balanced or unbiased, links to additional information, and identification of uncertainties) and quality (description of treatments and underlying mechanism, treatment benefits, treatment risks, likely progression with no treatment, alternative treatment options, impact of different treatments on quality of life, and support for shared decision-making), and provides detailed criteria by which to identify and assess each item.

To assess the cultural safety of osteoarthritis information, in the absence of an existing validated or widely used model or framework, we compiled a 5-item framework from the only available exploratory studies of barriers to or preferences for culturally safe specific to osteoarthritis care among ethnoculturally diverse women: use of plain language (no medical jargon, use of active voice, brief uncomplicated sentences, and words less than 3 syllables), availability of translated versions, empathetic communication (language or tone conveys respect, understanding, and support; language avoids blame and does not provoke fear), recognition of ethnoculturally diverse beliefs and norms (depicts ethnoculturally diverse images, acknowledges different cultures, and uses cultural idioms), and strategies to overcome barriers of access to care [35-41].

#### Health Care Professionals

To extract data on quality, in the absence of an existing validated or widely used framework, we compiled a 7-item framework from the only available studies that identified quality criteria for online or virtual continuing education given that we wished to assess the quality of online continuing education about osteoarthritis: accommodated individual preferences for learning pace, content could be tailored to meet individual learning needs, provided example cases, included interactive components, evidence-based, supported skill enhancement, developed by credible sources and provided choice of format [4,42,43]. To assess cultural safety, in the absence of an existing validated or widely used framework, we compiled a 3-item framework from the only available exploratory studies of what constitutes cultural competency when caring for ethnoculturally diverse persons with any type of health care concern: recognition of disparities in access to or quality of care, insight on ethnocultural beliefs or norms, and strategies to deliver culturally safe care [36,44-46].

### Data Analysis

SM and SD independently scored all osteoarthritis materials, then met to resolve discrepancies through discussion. ARG resolved the remaining uncertainties, and discrepancies, and checked all data. DISCERN scoring involved a 5-point scale with each of the 15 items marked as met (5), partially met (2-4), or not met (1). We deemed items that were not relevant to the materials as not applicable and did not record a score (eg, not all items included treatment information). We applied a similar scoring system for all other frameworks. For all instruments and frameworks, we derived an overall score based on the total number of “met” items divided by the total number of applicable items. Applying the same rubric as DISCERN, we categorized the overall score as low (<50%), moderate (50%-69%), or high (≥70%+). We used summary statistics, referring to simple counts of numbers and percentages, to report osteoarthritis material characteristics, design, quality, and cultural safety, and used tables to summarize data along with specific examples of osteoarthritis materials.

### Ethical Considerations

No ethics approval was required because all data were publicly available.

## Results

### Search Results

We identified 176 potentially eligible items: 122 for persons with osteoarthritis and 54 for health care professionals. We excluded 129 items that did not meet eligibility criteria. This included 79 items for persons with osteoarthritis: content not relevant to osteoarthritis (n=29), duplicate (n=27), no educational content (n=17), focused solely on joint replacement (n=5) or not produced in Canada (n=1); and 50 items for health care professionals: not relevant to osteoarthritis (n=23), blank template or form to record details for patient records (n=10), duplicate (n=8), clinical guideline or standard (n=5), or focused solely on joint replacement (n=4). Ultimately, we included 47 items: 43 for persons with osteoarthritis [47-89] and 4 for health care professionals [90-93].

### Characteristics

Multimedia Appendices 2 and 3 provide data on the characteristics of included osteoarthritis materials for persons with osteoarthritis and for health care professionals, respectively. Table 1 summarizes the characteristics of the included items.

**Table 1:** Summary of characteristics, quality, and cultural safety of 47 included osteoarthritis materials.

Year [reference]		Developer type	Development approach	Osteoarthritis topic	Format	Quality	Cultural safety
**Persons with osteoarthritis**							
	2023 [47]	Patient advocacy	Expert advice	General	Infographic	Low	Low
	2023 [48]	Charity	Not reported	General	Text + videos	Moderate	Moderate
	2023 [49]	Charity	Not reported	General	Infographic	Low	Low
	2023 [50]	Charity	Not reported	General	Text + videos	Moderate	High
	2023 [51]	Charity	Not reported	General	Checklist	Moderate	Moderate
	2023 [52]	Charity	Not reported	General	Survey + response summary	Moderate	Moderate
	2023 [53]	Charity	Not reported	General	Survey	Moderate	Moderate
	2023 [54]	Charity	Expert advice	General	Survey + response summary	Moderate	Low
	2023 [55]	Charity	Review	General	Survey	High	Low
	2023 [56]	Charity	Expert advice	General	Text	Moderate	Low
	2023 [57]	Charity	Expert advice	Hip or knee	Booklet	Moderate	High
	2023 [58]	Charity	Not reported	General	Infographic	Low	Moderate
	2023 [59]	Patient advocacy	Not reported	General	Survey + response summary	High	Low
	2023 [60]	Patient advocacy	Key informant interviews	General	Infographic	Low	High
	2022 [61]	Charity	Expert advice	General	Video	Low	High
	2022 [62]	Consortium	Not reported	Hip or knee	Text	Moderate	High
	2021 [63]	Charity	Expert advice	General	Text	Moderate	Moderate
	2021 [64]	Charity	Expert advice	General	Text	High	Moderate
	2021 [65]	Charity	Expert advice	General	Text	Moderate	Low
	2021 [66]	Patient advocacy	Review + expert advice	General	Video	Low	Moderate
	2020 [67]	Consortium	Not reported	General	Booklet	Moderate	High
	2020 [68]	Charity	Expert advice	Hip or knee + shoulder	Video	Moderate	Moderate
	2020 [69]	Charity	Expert advice	General	Video	Low	Low
	2020 [70]	Charity	Expert advice	General	Booklet	Moderate	High
	2020 [71]	Charity	Expert advice	General	Text	Low	Low
	2020 [72]	Charity	Expert advice	General	Text	Moderate	Low
	2019 [73]	Charity	Review + expert advice	General	Booklet	High	Low
	2019 [74]	Consortium	Not reported	General	Booklet	Moderate	Moderate
	2019 [75]	Academia	Expert advice	Hip or knee	Video	Moderate	High
	2018 [76]	Charity	Review	General	Booklet	High	Moderate
	2018 [77]	Charity	Review	General	Booklet	High	Moderate
	2018 [78]	Charity	Review	General	Booklet	High	Moderate
	2018 [79]	Charity	Review	General	Booklet	High	Moderate
	2018 [80]	Charity	Review	General	Booklet	High	Moderate
	2018 [81]	Charity	Review	General	Booklet	High	High
	2018 [82]	Charity	Review	General	Booklet	High	Moderate
	2018 [83]	Charity	Review	General	Booklet	High	Moderate
	2018 [84]	Patient advocacy	Review + expert advice	General	Booklet	Moderate	Moderate
	2018 [85]	Government	Expert advice	Hip or knee + hand	Booklet	Moderate	High
	2018 [86]	Charity	Not reported	General	Checklist	Low	Moderate
	2018 [87]	Government	Not reported	General	Text	High	High
	2015 [88]	Charity	Not reported	General	Checklist	Moderate	Moderate
	2013 [89]	Consortium	Expert advice	General	Infographic	Low	Moderate
**Health care professionals**							
	2022 [90]	Charity	Expert advice	General	Infographic	Moderate	Low
	2022 [91]	Consortium	Expert advice	Knee	Text	Low	Low
	2020 [92]	Professional society	Review	General	Infographic	High	Low
	2017 [93]	Consortium	Expert advice	Hip or knee + hand	Infographic	High	Low

#### Persons With Osteoarthritis

In total, 43 materials for persons with osteoarthritis were published between 2013 and 2023 (median 2020). Most were developed by charities (31/43, 72.1%) followed by patient advocacy groups (5/43, 11.6%), consortia (4/43, 9.3%), government (2/43, 4.7%), and academia (1/43, 2.3%). A total of 32.6% (14/43) of materials did not report development methods. Of the remaining 29 that did, most (16, 55.2%) were based on expert advice, 9 (31%) on review of published research, 3 (10.3%) on review of published research and expert advice, and 1 (3.4%) on key informant interviews. The majority (38/43, 88.4%) of materials focused on osteoarthritis in general while 11.6% (5/43) focused on hip and knee osteoarthritis with or without hand or shoulder osteoarthritis. With respect to format, the largest proportion were booklets (15/43, 34.9%) followed by text on web pages with or without other items such as videos (10/43, 23.3%), infographics (5/43, 11.6%), surveys with or without response summaries (5/43, 11.6%), and checklists (3/43, 7%).

#### Health Care Professionals

A total of 4 materials for health care professionals were published between 2017 and 2022 by consortia (n=2, 50%), a charity (n=1, 25%), and a professional society (n=1, 25%). Methods of development included expert advice (3/4, 75%) and review of published research (1/4, 25%). In total, 50% (2/4) of materials focused on osteoarthritis in general and 50% (2/4) on knee osteoarthritis with or without hip or hand osteoarthritis. A total of 75% (3/4) of materials were infographics, and 25% (1/4) of item consisted of text on web pages.

### Quality

Multimedia Appendices 4 and 5 provide data on the quality of included osteoarthritis materials for persons with osteoarthritis and for health care professionals, respectively. Table 1 summarizes the quality of the included items. There did not appear to be associations between osteoarthritis material quality and other characteristics.

#### Persons With Osteoarthritis

Of the 43 materials for persons with osteoarthritis, 23.3% (n=10), 46.5% (n=20), and 30.2% (n=13) were rated as low, moderate, and high on the DISCERN scale for quality, respectively. An example of a low-scoring item was “What is osteoarthritis” with a score of 26.7% [49]. As a one-page infographic, details about symptoms, diagnosis, and treatment are sparse, with little to no detail about sources of the information, accounting for only 8 of 15 DISCERN criteria that were fully met. An example of a high-scoring item was “Daily living online learning module” with a score of 86.7% [78]. As a 22-page booklet, this item fully met 13 of 15 DISCERN criteria by providing detailed advice on how to achieve a wide range of daily activities and links to sources of information.

#### Health Care Professionals

Of the 4 materials for health care professionals, 1 (25%) scored low, 1 moderate (25%), and 2 (50%) high on the framework for quality. The low-scoring item, “Knee’d: What to tell patients about knee injections for osteoarthritis”, presented text information on a web page and met only 2 (self-directed and needs-based) of 8 criteria for a score of 25% [91]. An example of a high-scoring item was the “Osteoarthritis tool” with a score of 87.5% [93]. This 8-page infographic met 7 of 8 criteria (self-directed, needs-based, evidence-based, interactive, support for practice, credible, and choice of format), but was not case-based.

### Cultural Safety

Multimedia Appendices 6 and 7 provide data on the cultural safety of included osteoarthritis materials for persons with osteoarthritis and for health care professionals, respectively. Table 1 summarizes the cultural safety of the included items. There did not appear to be associations between osteoarthritis material cultural safety and characteristics.

#### Persons With Osteoarthritis

Of the 43 materials for persons with osteoarthritis, 11 (25.6%), 21 (48.8%), and 11 (25.6%) were rated as low, moderate, and high cultural safety, respectively. An example of a low cultural safety item was “How Can An OT Help People With Arthritis?” [47]. This 1-page infographic met 2 criteria (plain language and empathetic communication) but did not address cultural beliefs and norms or offer solutions to overcome barriers and was not available in languages other than English. An example of a high cultural safety item was “Tips On How To Manage Daily Cooking Tasks and Live Well With Arthritis” with a score of 80% [60]. This 2-part infographic met 4 of 5 criteria (plain language, empathetic communication, accommodation of cultural beliefs and norms, and solutions to overcome barriers) but was not translated to languages other than English.

#### Health Care Professionals

Of the 4 materials for health care professionals, all (100%) were rated low for cultural safety. For example, “Conservative OA Treatments–Examples for Providers,” a 2-page infographic, did not acknowledge disparities in access to or quality of osteoarthritis care, identify related cultural issues or offer strategies to address cultural beliefs and norms [90].

## Discussion

### Principal Findings

We assessed the quality and cultural safety of 47 osteoarthritis materials: 43 for persons with osteoarthritis [47-89] and 4 for health care professionals [90-93]. Most were developed by charities, focused on osteoarthritis in general rather than osteoarthritis affecting a specific body part, and in the format of booklets. Many did not describe how they were developed; those that did were most often based on expert advice. Only 30% (13) patient osteoarthritis materials and 50% (2) health care professional materials scored high on the DISCERN scale for quality. Only 26% (11) of patient osteoarthritis materials scored high for cultural safety, and all health care professional osteoarthritis materials scored low for cultural safety. There did not appear to be associations between osteoarthritis material quality or cultural safety with other characteristics.

### Strengths and Limitations

This study featured several strengths. We used rigorous content analysis methods and complied with qualitative research reporting criteria [25-27]. To ensure reliability, multiple individuals independently performed searching and screening, and data extraction and analysis. We also used multiple frameworks or instruments to describe osteoarthritis material characteristics [33], quality [4,34,42,43], and cultural safety [35-41,44-46]. An interdisciplinary research team that included ethno-culturally diverse women with osteoarthritis guided all aspects of the study. We must also acknowledge several limitations. The search strategy may not have identified all relevant osteoarthritis materials. Also, because we assessed only publicly available osteoarthritis materials, we may have missed osteoarthritis materials available to health care professionals through individual or organizational subscriptions. We excluded osteoarthritis materials available only in French, Canada’s second official language. While we included only English-language materials, we assessed whether those materials were available in languages other than English, which was not the case for any included in this study. We included only osteoarthritis materials developed in Canada. This enhanced study feasibility, but possibly excluded osteoarthritis materials of high quality or cultural safety that may be widely accessible on the internet. However, others could use the frameworks and instruments used here to assess osteoarthritis materials developed in their own countries. With no validated instruments to assess the quality of online materials for health care professionals, and no validated instruments to assess cultural safety of materials for either patients or health care professionals, we relied on sparse available research to compile frameworks by which to evaluate included materials. Future research could expand on and validate these frameworks.

### Comparison With Previous Work

Little previous research on support for first-line osteoarthritis management examined the characteristics of online osteoarthritis materials for patients or health care professionals [22-24]. One study that assessed 125 web pages on shoulder arthroplasty reported a low mean of 51.4 (SD 10.7) and a median of 51.5 (IQR 30.5-71.5) DISCERN score [94]. However, the study did not specify that arthroplasty was specific to second-line therapy for advanced osteoarthritis, and did not report which materials were for patients or health care professionals [94]. A meta-analysis of 8 randomized controlled trials showed that digital (eg, telephone, video, internet, and mobile app) self-management programs for persons with osteoarthritis significantly reduced pain and improved physical function at 12 months [95]. While this meta-analysis underscores the benefit of online osteoarthritis materials, it did not assess the quality or cultural safety of those materials. Similarly, another review of 11 randomized controlled trials found that digital (eg, internet or telephone app) self-management support programs for persons with osteoarthritis ranged from 6 to 9 months in duration, largely focused on physical activity, and resulted in program adherence of >70% in studies that reported adherence but did not assess the quality or cultural safety of programs [96]. A systematic review of 25 articles on mobile health technologies (mobile phones or other devices) and an assessment of 23 apps relevant to osteoarthritis self-management focused only on categorizing those technologies by role: self-management, decision support, or shared decision-making [97]. A previous review of online health information for the public found that most resources were of low quality but did not assess cultural safety [6]. Hence, this study is unique because we explicitly assessed online osteoarthritis materials for patients and health care professionals and found the majority of osteoarthritis materials scored low or moderate for quality and cultural safety.

### Implications for Practice and Research

Given the high prevalence of osteoarthritis and its debilitating effects [7,8], lack of access to osteoarthritis care among high-risk racialized immigrant women [11-18], lack of high quality, culturally safe osteoarthritis materials for persons with osteoarthritis or health care professionals revealed by this study, and pan-Canadian recommendations for improved access to publicly available osteoarthritis materials [20,21], greater efforts are needed to develop such materials. With respect to development methods, few osteoarthritis materials were explicitly based on published research, relying instead on expert advice, but it was not clear in all cases whether “experts” included persons with osteoarthritis. This knowledge could be applied to the improvement of existing osteoarthritis materials or the development of new materials. Because of the long-standing recognized importance of evidence-informed decision-making, the rubrics we used to assess quality (eg, DISCERN for patient materials and frameworks we compiled for health care professional material) included explicit identification of the underlying evidence and sources of data [4,34,42,43]. Furthermore, meaningfully engaging patients, family or caregivers, or members of the public in planning or developing health care policy, services, interventions, or tools is increasingly encouraged and common [98], and numerous resources are available to provide guidance on how to optimize their engagement [99]. Additional insight could be borrowed from methods used to engage persons with lived experience in developing other types of tools such as decision aids for patients and clinical guidelines for health care professionals [100,101]. Developers may want to give extra attention to quality domains that were frequently not met by osteoarthritis materials included in this study; for example, sources of information and benefits and harms of treatment for patient materials; and case-based and interactive for health care professional material. While we compiled cultural safety frameworks for both patient and health care professional osteoarthritis materials based on our research and that of others [35-41,44-46], none were specific to osteoarthritis, therefore further research is needed to establish what constitutes cultural safety in the context of osteoarthritis.

### Conclusions

We aimed to assess the quality and cultural safety of online osteoarthritis informational or educational materials for persons with osteoarthritis and health care professionals. Of the 47 osteoarthritis materials (43 for persons with osteoarthritis, 4 for health care professionals), most were developed by charities, focused on osteoarthritis in general rather than osteoarthritis affecting a specific body part, and in the format of booklets. Many did not describe how they were developed; those that did were most often based on expert advice rather than explicit evidence. Few osteoarthritis materials for persons with osteoarthritis or health care professionals scored high for quality. Few osteoarthritis materials for persons with osteoarthritis and none for health care professionals scored high for cultural safety. Quality and cultural safety did not appear to be associated with osteoarthritis material characteristics. These findings reveal the need for further efforts to improve existing or develop new osteoarthritis materials for both persons with osteoarthritis and health care professionals. Further research is needed to assess the quality and cultural safety of osteoarthritis materials developed by organizations outside of Canada and to establish a framework or instrument to assess cultural safety in the osteoarthritis context.

## Appendix

Multimedia Appendix 1 Eligibility criteria.

Multimedia Appendix 2 Characteristics of materials for persons with osteoarthritis.

Multimedia Appendix 3 Characteristics of materials for health care professionals.

Multimedia Appendix 4 Quality of materials for persons with osteoarthritis.

Multimedia Appendix 5 Quality of materials for health care professionals.

Multimedia Appendix 6 Cultural safety of materials for persons with osteoarthritis.

Multimedia Appendix 7 Cultural safety of materials for health care professionals.

## Data Availability

All data generated or analyzed during this study are included in the published article and supplementary information files.
