# Supplementary material for: The Quality and Cultural Safety of Online Osteoarthritis Information for Affected Persons and Health Care Professionals: Content Analysis
Source: J Med Internet Res. 2024 Oct 18;26:e57698. doi: 10.2196/57698 (PMC11530738; doi:10.2196/57698)
Supplement: Multimedia Appendix 1 [file jmir_v26i1e57698_app1.docx]

Multimedia Appendix 1: Eligibility criteria

| Inclusion category | Inclusion criteria | |
| --- | --- | --- |
|  | Persons with OA | Healthcare professionals |
| Recipients | For adults aged 18+ who are women or persons with OA in general with suspected or diagnosed OA  Not eligible:   - Information tailored for individuals below 18 years of age - Content intended for a demographic other than women or persons with osteoarthritis (OA) | Practicing healthcare professionals that offer first-line treatment including but not limited to: nurse practitioners, family physicians, physical therapists, physiotherapists, occupational therapists, chiropractors, registered massage therapists, exercise therapists or nutritionists  Not eligible:   - Resources primarily designed for non-healthcare roles (e.g., administrative staff) - Community-based professionals that provide assistance to persons with OA but who are not clinicians (e.g. pharmacists) |
| Personnel or setting | Material is provided by healthcare professionals or organizations of any type, or acquired directly by persons with OA from the developers or from other sources  Not eligible:   - Information co-created by members of a network or social group (e.g. online patient support group who offer advice to each other) | Material is provided to healthcare professionals by professional societies, or acquired directly by healthcare professionals from the developers or other sources  Not eligible:   - Settings other than those providing first-line care (e.g. long-term care homes) |
| Format | Including but not limited to the following:   - Video (including slides or narrated slides) - Infographic - information or data presented graphically with pictures or charts +/- text, of usually one or two pages - Checklist - list of items to know or practice; list of questions (i.e. question prompt tool to support communication), or list of activities, all +/- space to enter information - Booklet - multi-page document, usually more than two pages, featuring information +/- graphical elements - Web sites featuring text +/- any of the above formatted materials   Not eligible:   - Mobile apps - Any resources that require an account and/or payment to access information | SEE Persons with OA column |
| Developer | Canadian non-profit organizations such as government, governmental agency, academic group, professional society or disease-specific foundation  Not eligible:   - Information developed or produced by organizations outside of Canada | |
| Content | English language information including but not limited to OA or broader concept of joint inflammation, cause, prevention, diagnosis, and clinical and self-management, published or updated in 2012 or later to reflect recommendations in current clinical practice guidelines  Not eligible:   - Information in languages other than English - Information that solely focuses on second-line management, which includes surgery or joint replacement | |
| Delivery | Publicly-available on the Internet to be viewed on web pages or online files, or printed or downloaded to other devices  Not eligible:   - Clinical practice guidelines; however, we will include educational material included in or with guidelines that meets these eligibility criteria | |
| Timing | Viewed in print or electronic format on one or more occasions as desired | |
