# Supplementary material for: The Quality and Cultural Safety of Online Osteoarthritis Information for Affected Persons and Health Care Professionals: Content Analysis
Source: J Med Internet Res. 2024 Oct 18;26:e57698. doi: 10.2196/57698 (PMC11530738; doi:10.2196/57698)
Supplement: Multimedia Appendix 2 [file jmir_v26i1e57698_app2.docx]

Multimedia Appendix 2. Characteristics of included educational materials for persons with osteoarthritis

| Material | | | Characteristics | | |
| --- | --- | --- | --- | --- | --- |
| Title Developer Year published  [Reference]  Web address | Developer type  Approach | Objective  Type of OA | Content | Format  Pages (n) or Duration (min) | Delivery |
| How Can An OT Help People With Arthritis?  Canadian Arthritis Patient Alliance  2023[47]  <https://arthritispatient.ca/wp-content/uploads/2023/09/Occupational-Therapy-infographic.pdf> | Patient advocacy  Expert advice | Describe OA self- Management  General OA | Eight ways in which occupational therapists (OTs) can assist persons living with arthritis include   1. Recommend assistive technologies like right angled knife, split keyboard, furniture riser etc. 2. Prescribe mobility aids such as canes, walkers and wheelchairs 3. Suggest strategies to help save energy like pacing, positioning, planning and prioritizing 4. Educate about pain-fatigue-mood relationship, cognitive behavioural therapy, mindfulness and energy conservation 5. Create custom fabricated splint or recommend off the shelf options 6. Prescribe exercises 7. Suggest strategies to promote better sleep   Assess safety of home and recommend home modification to complete regular activities | Infographics  1 page | Available online to read or download |
| Osteoarthritis  Arthritis Society Canada  2023 [48]  <https://arthritis.ca/about-arthritis/arthritis-types-(a-z)/types/osteoarthritis> | Charity  Not reported | Describe OA symptoms, diagnosis, treatment and self-management  General OA | Introduction to OA  Example of a healthy joint and a joint with OA  Links to additional web-resources  Ten resources: (1) Symptom and diagnosis (2) Self-Management (3) Treatment (4) resources for clinicians (5) Managing pain (6) Staying active(7) Eating well (8) View more guides (9) Arthritis Talks webinars –three videos about Complementary therapies and emerging treatments, strategies to manage your pain and delayed surgery and arthritis pain (10) View more webinars | Information organized by topic sub-headings and three videos of 60 minutes | Information available to view on multiple web pages  and videos available on YouTube to view by streaming |
| What Is Osteoarthritis?  Arthritis Society Canada  2023 [49]  <https://arthritis-society.azureedge.net/media/as/media/pdf/whatisoa_infographic.pdf> | Charity  Not reported | Describe OA symptoms, diagnosis, risk factor, self management and treatment  General OA | Brief OA introduction with diagrammatic representation of OA symptoms (joint pain, swelling, difficulty moving, stiffness, grinding or creaking noise, unstable joint)  Risk factors associated with OA include overweight, differently shaped joints, joint injury, repeated stressful activity and low physical activity, and method of diagnosis through symptoms, physical exam and x ray.  Self-management strategies include exercise, weight management, heat and cold treatment, assistive devices, mindfulness meditation and avoiding lifting.  List of treatment include physiotherapy and occupational therapy, medications and surgery  Links to additional web resources  Two resources :(1) Arthritis symptom checker and (2) Arthritis.ca/osteoarthritis | Infographic  1 page | Available online to read or download |
| Complementary Therapies Guide For Arthritis  Arthritis Society Canada  2023[50]  <https://arthritis.ca/treatment/pain-management/complementary-therapies> | Charity  Not reported | Describe OA treatment  General OA | Three cards that outline an introduction to complementary therapy, delineate various types, and offer guidance on selecting the most suitable complementary therapies for arthritis  A video that discusses:   - Exercises such as Tai Chi and Qigong - Supplements such as turmeric, echinacea and ginger - Diets such as gluten-free, low-FODMAP and Neolithic - Emerging treatments such as cell therapies and medical cannabis, and - Complementary therapies such as massage therapy, meditation and acupuncture   Links to additional web-resources  Three resources: (1) Drug-Free pain management tool and (2) Online learning Managing chronic Pain and (3) Flourish | Information organized by topic sub-headings and a 60 minutes video | Information available to view on multiple web pages  and video is available on YouTube to view by streaming |
| Appointment Discussion Guide  Arthritis Society Canada  2023 [51]  <https://arthritis.ca/getmedia/d595f5ae-688e-44a0-8b7b-01ce2dadc698/AppointmentDiscussionGuide_EN_Jan10B.pdf> | Charity  Not reported | Help persons with OA to prepare for healthcare appointments  General OA | List of 10 questions to consider discussing during appointment with health care provider:   1. How long have you been having pain, discomfort, swelling, inflammation, or other symptoms affecting your joints? List out all symptoms you have been experiencing. 2. Have you had any recent injuries that have affected your joints or joint movement? 3. Are you currently experiencing pain? If so, how would you rate your pain on a scale from 1-10, with 1 being no pain, and 10 being the worst pain you can imagine? 4. Does your pain or other symptom(s) come and go, or is it constant? 5. Does your pain level change with activities or movement? What makes it better, or worse? 6. Have you taken any medication for your pain or other symptoms (over the counter, prescription, or natural products)? If so, what have you taken, and was it helpful? Did it raise or lower your pain level? By how much? list all the medications you have taken. 7. What level of impact do your symptoms have in your daily activities? Have you had to change any part of your daily routine because of pain, discomfort, or a change in physical ability? 8. Have you used massage, physical therapy, or another complementary health treatment to manage your symptoms? If so, how did it affect your symptoms? 9. Have your symptoms affected your mood, energy levels, or other aspects of your life? 10. To your knowledge, does anyone else in your family have arthritis? If so, do you know what kind?   Links to additional web resources  Three resources: (1) Medication Tracker sheet (2) Symptom checker and (3) Patient journey | Checklist  1 page | Available online to read or download |
| Arthritis Symptom Checker  Arthritis Society Canada  2023 [52]  <https://arthritis.ca/about-arthritis/signs-of-arthritis/symptom-checker/> | Charity  Expert advice: Committee of  orthopaedic surgeons and persons with OA | Help persons with OA to prepare for healthcare appointments  General OA | Questions about pain persistence, joints affected (symmetrical or asymmetrical) pain duration and symptoms to self-assess whether a person has OA.  Upon submitting responses to all questions, a summary of responses appears  Links to additional web-resources  Two resources: (1) Prepare for your appointment and (2) Start taking action now | Survey with 6 questions, summary of responses and potential diagnosis | Multiple web pages. Summary can be read online, or emailed or printed |
| Daily Symptom Tracker  Arthritis Society Canada  2023[53]  <https://arthritis.ca/AS/media/pdf/Support%20and%20Education/EN-daily-symptom-tracker.pdf> | Charity  Not reported | Help persons with OA to prepare for healthcare appointments  General OA | List of symptoms include pain, fatigue, mood, stress, physical activity/exercise, healthy eating and social life and scale of 1 to 5 to rate them to identify their pattern  Space to note any out of routine activities for example activities, medications, treatment, overall health etc. | Survey with 7 questions | Available online to read or download |
| Arthritis Risk Factor Assessment  Arthritis Society Canada  2023[54]  <https://arthritis.ca/about-arthritis/arthritis-risk-factors/arthritis-risk-factor-assessment> | Charity  Expert advice: Committee of  orthopaedic surgeons and persons with OA | Evaluate OA risk factors  General OA | Set of specific questions about age, sex, body weight, genetic factors, and smoking habits, history of injury, physical activity level, diet, and susceptibility to infection to comprehensively evaluate and assess potential risk factors for OA  Upon submitting each responses description of risk related to response appears | Survey with 10 questions and summary of risk factors | Multiple webpage |
| Joint Pain Symptom Checker  Arthritis Society Canada  2023[55]  <https://arthritis.ca/getmedia/b4fac3b9-8b48-4ec1-ac38-6bba4e2f2322/EN-joint-symptom-tracker-2023.pdf> | Charity  Review of published research | Help persons with OA to prepare for healthcare appointments  General OA | List of questions detailing joint pain symptoms, providing options to choose "Yes" or "No" based on the presence of symptoms, for self-assessment and monitoring. | Survey with 21 question | Available online to read or download |
| Medication Reference Guide  Arthritis Society Canada  2023[56]  <https://arthritis.ca/treatment/medication/medication-reference-guide?atype=694c7b0f-c2fb-4afc-be72-0b4924263294&atype> | Charity  Expert advice: Pharmacists | Describe OA medication  General OA | Different type of OA medication categorized by drug class.  List of drug include Duloxetine, Non-steroidal Anti-inflammatory Drugs, Steroid injection and viscosupplementation.  Information on brand name, drug use ,mode of administration, doses, mode of action, contraindication, side effects, storage method and if the medications are covered under any patient support program | Information organized by topic sub-headings | Multiple webpages |
| Low Load Activities For Osteoarthritis  Arthritis Society Canada  2023[57]  <https://arthritis.ca/getmedia/28589053-8d9d-4860-892c-b236e5ced8b3/LowLoad_Activities_EN-Jun1_small.pdf> | Charity  Expert advice: Physiotherapist | Describe OA exercises  Hip and knee OA | Activities that put less weight load on the joints that includes cycling, walking with poles, tai chi, yoga, pilates, and water-based activities like aqua fit, swimming, or pool walking. | Booklet  2 pages | Available online to read or download |
| Modifying Activities For Osteoarthritis  Arthritis Society Canada  2023[58]  <https://arthritis.ca/getmedia/a142085b-aa62-4842-be5a-41a972c4fd44/Activity_Modifications_EN-Jan20_small.pdf> | Charity  Not reported | Describe OA exercises  General OA | Activities that may cause pain include high impact, twisting, forceful end of movement, getting down to the floor and up and kneeling and ways to modify them. For example use body weight to push down to reduce high impact, use towel or yoga block to limit bending, use gardening bench or stool to avoid getting up and down on the floor, use knee pads when kneeling to avoid knee pain etc. | Infographic  1 page | Available online to read or download |
| Arthritis Screening Exam  Arthritis Consumer expert and Arthritis research Canada  2023 [59]  <https://arthritisbroadcastnetwork.org/arthritis-screening/> | Patient advocacy  Not reported | Help persons with OA to prepare for healthcare appointments  General OA | Image to select areas and joints that are swollen or painful  List of following questions   1. Does a joint(s) hurt? 2. Is the main problem your back? 3. When you get out of bed are your joints stiff for more than one hour? 4. Is the pain worse when you stand or walk? 5. Does the pain wake you up most nights after you have gone to sleep? 6. Is the pain better if you move around a little? 7. Have you become a lot more tired since the joint pain started? 8. Do you, a parent or a brother or sister have psoriasis? 9. Do you get a skin rash on sun exposure, dead white pallor of the digits on cold exposure (Raynaud's) or ulcers inside the mouth every month?   Upon submitting responses to all questions, potential diagnosis appears with an online link to the resulting arthritis type | Survey with 9 questions, and result with potential diagnosis | Multiple web pages. Summary can be read online, or emailed or printed |
| Tips On How To Manage Daily Cooking Tasks and Live Well With Arthritis  Canadian Arthritis Patient Alliance  2023[60]  <https://arthritispatient.ca/tips-on-how-to-manage-daily-cooking-tasks-and-live-well-with-arthritis/> | Patient Advocacy  Interview with key informants | Describe OA self-management  General OA | Tips for living well with arthritis include   1. Be kind to yourself 2. Listen to your body 3. Stay active 4. Join a community and get involved 5. Advocate for yourself   Tips for cooking with arthritis include  Maintain position and posture, watch pace and take rest, keep track of nutrition, meal preparation in batches, use of gadgets to open jars and tabs, use lighter tools, use right angled knives for easy grip, use alternative appliances like toaster, air fryers etc., use of dycem squares so that materials don’t slip. | Information organized as two infographics | Single webpage. Infographic available online to read or download |
| Tai Chi To Help Arthritis  Arthritis Society Canada  2022[61]  <https://www.youtube.com/watch?v=qj0ft1CQC_4> | Charity  Expert advice: Physician | Describe OA exercise  General OA | Tai Chi exercises to improve strength, balance and flexibility   - Introduction to Tai Chi - Tai Chi breathing - The Dang Tian breathing method - Warm up exercises - Cool down exercises - Stretches of neck, spine and knee - Cooling tank exercise | Video  82 minutes | Available on YouTube to view by streaming |
| Osteoarthritis  GLA: D Canada  2022[62]  <https://gladcanada.ca/osteoarthritis> | Consortium  Not reported | Describe OA symptoms, diagnosis, treatment and self-management  Hip and Knee OA | Example of a healthy joint and a joint with OA  Distinctive symptoms associated with OA in the knees, hips, and hands  Key signs indicative of OA elaborating on how OA is diagnosed  Treatment options include self-management through exercise, weight management, pharmacologic and non-pharmacologic pain relief, therapist treatment, assistive devices or surgery for a spectrum of OA symptoms, from mild to severe. | Information organized by topic sub-headings | Multiple web pages |
| Osteoarthritis Symptoms And Diagnosis  Arthritis Society Canada  2021[63]  <https://arthritis.ca/about-arthritis/arthritis-types-(a-z)/types/osteoarthritis/osteoarthritis-symptoms-and-diagnosis> | Charity  Expert advice: Committee of  orthopaedic surgeons and persons with OA | Describe OA symptoms and diagnosis  General OA | Joints most commonly affected by OA are neck, shoulder, hands, hip, back, knees and foot  Common OA joint symptoms that include pain, stiffness, swelling, crepitus, instability and dysfunction and OA symptoms outside the joint include sleep disruption, altered mood, fatigue, quality of life and pain sensitization  Risk factor for OA include age, sex, family history, excess body weight, joint injury, occupations, joint deformity, sedentary lifestyle  Diagnosis of OA through symptoms, physical examination, X-ray and MRI  OA patient journey from symptoms to diagnosis and later to treatment and management  Links to additional web-resources:  Four resources: (1) Arthritis symptom checker (2) Talk to your doctor about your joint pain (3) Arthritis risk assessment and (4) Osteoarthritis Patient journey | Information organized by topic sub-headings | Multiple web pages |
| Osteoarthritis Self-Management  Arthritis Society Canada  2021[64]  <https://arthritis.ca/about-arthritis/arthritis-types-(a-z)/types/osteoarthritis/osteoarthritis-self-management> | Charity  Expert advice: Committee of  orthopaedic surgeons and persons with OA | Describe OA self-management  General OA | Self-management strategies such as physical activities, protect your joint, effective weight management, heat and cold therapy and relaxation and coping skills  Physical activity include therapeutic exercise, range of motion exercise, strengthening exercise and endurance exercise  Protect your joint include techniques that help to protect joint. For example: pace yourself, keep joint aligned, avoid excess joint stress, see a physical or occupational therapist and use assistive devices  Effective weight management include ways to improve nutrition such as limit saturated and trans fats, reduce sugar intake and eat more vegetables and fruit, supplements like vitamin D, glucosamine, chondroitin and turmeric  Heat and cold therapy include taking hot shower, using warm or cold packs  Links to additional web-resources  Eighteen resources: (1) Free online arthritis self-management learning resources (2) Canadian 24-hour movement guidelines (3) Adaptive sports (4) Tai Chi to Help Arthritis video (5) 20-Minute Warm-Up for the Joints video (6) Flourish articles on Exercise & Motion (7) Exercises for osteoarthritis of the hip & knee (8) Exercises for osteoarthritis of the shoulder (9) Introduction to exercises for osteoarthritis (10) Low load activities for osteoarthritis (11) Modifying activities for osteoarthritis (12) Daily living learning guide (12) Can decreasing weight decrease pain (13) Eating well online learning guide (14) Managing Chronic pain online learning guide (15) Living your best life with osteoarthritis (16) A guide to living with Osteoarthritis (17) Canadian Arthritis Patient Alliance and (18) GLA:D | Information organized by topic sub-headings | Multiple webpages |
| Osteoarthritis Treatment  Arthritis Society Canada  2021[65]  <https://arthritis.ca/about-arthritis/arthritis-types-(a-z)/types/osteoarthritis/osteoarthritis-treatment> | Charity  Expert advice: Committee of  orthopaedic surgeons and persons with OA | Describe OA treatment  General OA | Treatment include therapies, medication and surgery  Example of therapies are physiotherapy, occupational therapy, Acupuncture and Massage  Medication list include topical treatments, corticosteroid injections, acetaminophen, non-steroidal anti-inflammatory drugs, Duloxetine and other injections, medical Cannabis and opioids  Surgery option like joint replacement  Links to additional web-resources  Five resources: (1) Assistive device resources (2) Drug –Free pain management tool (3) Complementary therapies guide for arthritis (4) Arthritis medication reference guide and (5) Surgery guide | Information organized by topic sub-headings | Multiple webpages |
| Updates In Osteoarthritis Treatment And Care With Dr. Tom Appleton  Joint health: Arthritis Consumer Experts  2021[66]  <https://arthritisathome.jointhealth.org/?p=2286> | Patient Advocacy  Expert advice: review of published research and expert advice from rheumatologists | Describe OA treatment  General OA | Latest research on physical activities for person with OA and update on clinical trial of medications for OA as below:   - Medication for treating OA - Monoclonal antibodies - Anti-nerve growth factor antibodies   Mapping out the future of virtual medicine in Canada | Video  16 minutes | Available on YouTube to view by streaming |
| Living Your Best Life With Osteoarthritis  Alberta Health Services,  Alberta Bone & Joint Health Institute  2020[67]  <https://www.albertahealthservices.ca/assets/about/scn/ahs-scn-bjh-osteoarthritis-toolkit.pdf> | Consortium (government + charity)  Not reported | Describe OA symptoms, diagnosis and self-management  General OA | Example of a healthy joint and a joint with OA and risk factors of OA  Self-management strategies like different exercises( cardio, strength exercise and range of motion exercise), healthy eating options, assistive devices, pain management medications and general and mental wellbeing that include managing stress, depression and anxiety, sleep, dental health and quitting smoking  Two Supporting tools: (1) Patient Report Card to track things one can try to manage OA (2) Treatment Menu for Patients Living with OA  Links to additional web resource  One web-resource: (1) Canadas food guide | Booklet  24 pages | Available online to read or download |
| Introduction To Exercise For Osteoarthritis  Arthritis Society Canada  2020[68]  <https://arthritis.ca/living-well/2020/introduction-to-exercise-for-osteoarthritis> | Charity  Expert advice: Physiotherapist | Describe OA exercises  Hip, knee and shoulder OA | Introduction to OA and exercises   - Difference between exercise and physical activity - Basic exercise principles - Barriers to physical activities muscles - Tips to get started   Exercises for OA of hip and Knee   - Starting an exercise program - Exercises for hips and abductor muscles - Exercises for quadriceps, hamstring and calf muscles - Exercises for trunk and core - Stretches   Exercises for OA of shoulder   - Introduction to exercises - Exercises for shoulder range of motion and stretching - Exercises for shoulder strengthening (rotation) - Exercises for shoulder strengthening (flexion, abduction and extension) - Exercises for scapular muscle stabilization | Three videos that range from 21 minutes to 47 minutes | Available on YouTube to view by streaming |
| 20-Minute Warm-Up For The Joints  Arthritis Society Canada  2020[69]  <https://www.youtube.com/watch?v=X15pa33QO-o&t=2s> | Charity  Expert advice: Yoga instructor | Describe OA exercises  General OA | 20 minutes warm up exercises for OA   - Introduction to warm up - Leg extension - Side bends - Gentle twist - Cat cow - Shoulder roll - Triceps and wrist stretches - Savasana | Video  21 minutes | Available on YouTube to view by streaming |
| OA: Patient Journey  Arthritis Society Canada  2020[70]  <https://arthritis.ca/getmedia/26457d6d-41cf-4c7f-8fbb-b26f75b88781/OA_Flowchart_March31_EN_small_interactive.pdf> | Charity  Expert advice: Committee of physician, occupational therapist, physiotherapist and orthopaedic surgeon | Describe OA symptoms, diagnosis self-management and treatment  General OA | Patient experiences include OA symptoms, interactions with healthcare providers, diagnosis, and the development of a care plan that integrates self-management strategies such as exercises, daily activity modification, and holistic wellness through weight management, mindful relaxation, and medication.  Treatment options include:   1. Pharmacological -topical creams, pain medication, injection and medical cannabis 2. Referral for surgery 3. Specialty care for therapeutic modalities, complementary therapies and psychological support   Links to additional web-resources  Five resources: (1) Risk assessment and (2) How to talk to your doctor about joint pain, (3) The Arthritis Society: arthritis.ca, Support & Education pages and YouTube channel (4) Healthy eating (5) GLA:D Canada | Booklet  2 pages | Available online to read or download |
| Assistive Devices Resource  Arthritis Society Canada  2020[71]  <https://arthritis.ca/treatment/pain-management/assistive-devices-resource> | Charity  Expert advice: Occupational therapist | Describe OA assistive devices  General OA | List of assistive devices and stores to buy those devices to help perform everyday tasks:   1. Bedroom-Zipper pull, button hook, Velcro, shoehorn, sock aids, bed risers 2. Kitchen- electric can opener, reacher, jar key, jar opener, adaptive handles and grips, non-slip grip 3. Bathroom- Handrails/grab bars, lever taps / door lever, raised toilet seat, bath seat, electric toothbrush, electric razor, detachable handheld shower head, small squeeze bottles/jars/tubes, toilet wiping aids, non-slip bath mat, lotion applicator 4. Office-Ergonomic chairs and desk and hands free headsets 5. Driving- key turner, gas cap opener, car transfer handle, wide-angled rear-view mirror / side mirror extension activities 6. Around the house- non-slip grip pen/writing grip, transfer handle, switch extender, non- slip doorknob covers and phone holder 7. Mobility device-Cane, walker and wheelchair 8. Activities- lightweight hoses, garden kneeler/seat, book holder, card holder and personal shopping cart/trolley | Information organized by topic sub-headings | Multiple web pages |
| Drug Free Pain Management Tool  Arthritis Society Canada  2020[72]  <https://arthritis.ca/treatment/pain-management/drug-free-pain-management-tool> | Charity  Expert advice: Committee of Physiotherapist and persons with OA | Describe OA self-management  General OA | Multiple pain management option categorized by arthritis type, treatment type that include all, self-management, complementary ,physiotherapy and occupational therapy with four different level of recommendation that encompass all type, high , medium and low  Management strategies include patient education, physical activity, weight loss, mindfulness, meditation and relaxation, Cognitive behavioural therapy, therapeutic writing , heat and cold therapy, physiotherapy management, balance training, electric stimulation, ultrasound therapy, cold laser therapy, manual therapy, therapeutic taping, joint protection, splint, return to work and work, assistive devices, biomechanics, biofeedback, using a cane, massage therapy, traditional Chinese medicine, acupuncture and Tai Chi | Information organized by filters sub-headings | Multiple webpages |
| Medical Cannabis And Arthritis  Arthritis Society Canada  2019[73]  <https://arthritis.ca/getmedia/f43c06f9-9e25-4524-a22a-18b1666d3d09/Medical-Cannabis-Guide-EN.pdf> | Charity  Review of published research and expert advice from rheumatologists | Describe OA treatment  General OA | Introduction to medical cannabis that include difference between medical and recreational cannabis ,its scientific foundations, forms of medical cannabis and applications in managing OA  Method of assessing and administering medical cannabis with potential side effects, effects on sleep, anxiety and appetite and associated risks  Links to additional web resources  Three resources: (1) Growing your own cannabis (2) Managing Chronic Pain and (3) Medical Cannabis Webpage | Booklet  43 pages | Available online to read or download |
| Talk To Your Doctor about Joint Pain  Arthritis Alliance of Canada  2019[74]  <https://arthritis.ca/getmedia/a2609338-226f-4bbe-8786-a4fd6f35f7ec/Talk_to_Your_Doctor_about_Joint_Pain_Handout_letter_EN> | Consortium  Not reported | Help persons with OA to prepare for healthcare appointments  General OA | List of common OA symptoms, associated risks and issues to share with doctor such as rate of pain, its impact in life and following questions:   1. Is my joint pain caused by osteoarthritis? 2. What can I do to help my symptoms of pain and stiffness? 3. What ways are proven to reduce my pain symptoms? 4. Do I have to take medicine or are there other things I can do to help myself? 5. How long will it take for the prescribed medication to take effect? 6. What can I do to help prevent further joint damage? 7. Should I exercise if I have joint pain? How much and how often? 8. What are reliable websites with more patient information on arthritis? 9. What to expect in joint examination and some self-management strategies like physical activities and their modification, diet and medication.   Link to additional web resources  Three resource: (1) Water based exercise (2) Patient resources websites and (3) Walking clubs | Booklet  4 pages | Available online to read or download |
| Exercises For Arthritis  University of Alberta  2019[75]  <https://www.ualberta.ca/glen-sather-clinic/patient-resources/exercises-for-arthritis.html> | Academia  Expert advice : Physical therapists | Describe OA exercises  Knee and Hip OA | Full COMET Exercises for arthritis   - Demonstration of quadriceps and gluteal muscles - Four phases of exercises categorized according to difficulty level - Instruction to four phases of exercises   Phase 1 video include four exercises:   - Quadriceps setting - Heel slide - Passive knee extension and - Inner range and quad activation   Phase 2 include four exercises:   - Seated knee extension - Straight leg raises - Glute bridge and - Chair squat   Phase 3 include five exercises:   - Glute bridge extension - Hamstring bridge - Clamshell - Side- lying hip adduction and - Body weight squat   Phase 4 include four exercises   - Splits squats - Step up - Hip hikes - Standing hip flexion | Four videos that range from 5 minutes to 19 minutes | Available on YouTube to view by streaming |
| Managing Chronic Pain Online Learning Module  Arthritis Society Canada  2018[76]  <https://arthritis.ca/getmedia/a70cd03b-e2e4-4ab9-bcc6-40ddecb9882d/Managing-Chronic-Pain-Final.pdf> | Charity  Review of published research | Describe self-management and treatment  General OA | Introduction to pain, pain cycle, managed and unmanaged pain and active and passive pain  Strategies to relive pain include coping with pain, heat and cold therapy, posture, muscle relaxation, visualization, distraction, meditation, physical therapy, occupational therapy, massage therapy, physical activity and exercise  Pain medication for both general and arthritis pain and safety tips to take medication  Types of support include emotional support , physical support and being informed  Links to additional web-resources  Eight resources: (1) Pain Australia (2) Mental health and wellbeing (3) Activity Diary (4) Daily living (5) Mindfulness meditation for chronic pain (6) Treat inflammatory arthritis (7) Medication guide and (8) Medical Cannabis | Booklet  30 pages | Available online to read or download |
| Overcoming Fatigue Online Learning Module  Arthritis Society Canada  2018[77]  <https://arthritis.ca/getmedia/175ca269-5551-42ad-8ab6-9d22bff0eea8/Overcoming-Fatigue-Final.pdf> | Charity  Review of published research | Describe OA symptom and self-management  General OA | Introduction to fatigue and arthritis  Self-management strategies to overcome fatigue include balancing activities, proper sleep, physical activities and exercises, healthier diet, dealing with depression and chronic pain management  Links to additional web-resources  Seven resources: (1) Fatigue self-test (2) Things that might help with fatigue (3) Activity Diary (4) A Walk through the forest music (5) sleep diary (6) Top 10 Exercises cheat sheet and (7) Canadian 24 hour movement guideline | Booklet  33 pages | Available online to read or download |
| Daily Living Online Learning Module  Arthritis Society Canada  2018[78]  <https://arthritis.ca/getmedia/9f3c6e33-9390-4ae7-9c70-64c786099a78/Daily-Living-Final.pdf> | Charity  Review of published research | Describe OA self-management  General OA | Tips for everyday activities like choosing footwear, quitting smoking, driving , standing and lifting, getting intimate, using arthritis tools, devices, life hacks and use of splints.  Links to additional web-resources  Three resources: (1) Pedorthic Association of Canada (2) Finding the right chair for you and (3) Mental health and well being | Booklet  22 pages | Available online to read or download |
| Mental Health & Well-being Online Learning Module  Arthritis Society Canada  2018[79]  <https://arthritis.ca/getmedia/1c7b3e72-cc2e-4dbd-b571-2c7482a8d849/Mental-Health-and-Wellbeing-Final.pdf> | Charity  Review of published research | Describe OA self-management  General OA | Introduction to mental health and link between arthritis and mental health  Ways to deal with stress: stress busting and taking action against stress  Ways to take care of mental health include strategies for self-management, improving mental health, relationship between food and mood, methods for experimenting with and tracking the impact of adding or eliminating certain foods as well as role of nutrients in fighting depression.  Advice on what to drink, connection between physical activity and mood, tools for examining thought patterns, fostering positive self-talk, and addressing harmful thought patterns through resources like the emotional Event Worksheet, techniques for planning, prioritizing, pacing, and problem-solving. Relaxation techniques, including breathing exercises, meditation, mindfulness, and Progressive Muscular Relaxation (PMR) and tips for improving sleep.  Links to additional web-resources  Twenty resources: (1) The University of Michigan Depression Centre quick assessment tools (2) What is your stress index? (3) Canadian Mental Health Association Mental health for life Brochure (4) Eating well (5) Food and mood tracker (6) Staying active (7) Thinking realistically (8) Emotional event worksheet (9) Positive coping with health conditions (10) The antidepressant skills workbook (11) Dealing with depression (12) Overcoming fatigue (13) Mindfulness Mediation for Chronic Pain (14) Reflections and Guidance on the Cultivation of Mindfulness, (15) the mindfulness solution (16) how to get better sleep (17) Arthritis and work (18) Navigating your health care (19) Find a psychologist and (20) Find community mental health organizations | Booklet  62 pages | Available online to read or download |
| Arthritis and Work Online Learning Module  Arthritis Society Canada  2018[80]  <https://arthritis.ca/getmedia/0f374d04-eede-449d-99be-993a7d17b0f1/Arthritis-and-Work-Final.pdf> | Charity  Review of published research | Describe OA self-management  General OA | Relation between arthritis and work  Interpersonal and emotional challenges in the workplace and top strategies for dealing with OA at work such as talking to expert, planning work schedule, evaluating self-energy etc.  Tips to position body at work while sitting, standing, lifting and driving with tips to exercise at work  Benefit of disclosing about arthritis at work and requests to put in as accommodations like flexible work hours, equipment changes, task and timing adaptation, work from home etc.  Links to additional web-resources  Three resources: (1) Activity Diary, (2) Daily living (3) Link to a Provincial and territorial office in Alberta, Nova scotia, British Colombia, Nunavut, Manitoba, Ontario, New Brunswick, Prince Edward Island, New Found land and Labrador, Quebec, Northwest Territories, Saskatchewan and Yukon | Booklet  27 Pages | Available online to read or download |
| Staying Active Online Learning Module  Arthritis Society Canada  2018[81]  <https://arthritis.ca/getmedia/743fa1e2-a69d-4e5b-91c1-26e7748593cc/Staying-Active-Final.pdf> | Charity  Review of published research | Describe OA self- management  General OA | Example of a healthy joint and a joint with OA  Benefits of OA exercises and different types of  exercises for flexibility (range of motion and stretching) strength and endurance and  OA friendly exercises like walking, yoga, water exercise, cycling and tai chi.  Ways to set SMARTER goals for OA exercises  Specific- set up physical activity following Canada physical activity guideline  Measurable-Plan to increase activity gradually and keep track  Attainable-set a goal that your health allows  Relevant- set goal that you enjoy  Time limited-set date to achieve goal  Evaluate-track progress  Reward –celebrate success  Ways to overcome barrier when in pain, tired, bad weather, lack of time, cannot afford gym membership, boring activities, scared of failing  List of exercise that can be done at work that include ankle circles, heel lift, knee raises, leg lift, shoulder stretches, forward arm reaches, shoulder squeeze, finger walk, hip and calf stretch and walking  Links to additional web-resources  Three resources: (1) Canada’s Physical activity guide-National physical activity plan  (2) Government of Canada get active tip sheets and (3) Canadian physical activity guideline | Booklet  30 pages | Available online to read or download |
| Eating Well Online Learning Module  Arthritis Society Canada  2018[82]  <https://arthritis.ca/getmedia/3a67574b-d006-420e-9cbf-cb85d4ef613f/Eating-Well-Final.pdf> | Charity  Review of published research | Provide dietary guidance  General OA | 8 articles that describes how individual eats matters, experiment and keep track, food and mood, general and specific guidelines on what should a OA patient eat and drink with information on supplements(glucosamine, Omega-3 fatty acids, avocado soybean unsaponifiables, Vitamin D etc.)  Links to additional web resources  Four webpages: (1) Food and mood tracker (2) Daily symptom tracker (2) Canada’s Food guide (3) Health guide and (4) Flourish e-newsletter | Booklet  17 Pages | Available online to read or download |
| Navigating Your Healthcare online Learning Module  Arthritis Society Canada  2018[83]  <https://arthritis.ca/getmedia/0aaa21d2-bf16-472e-b96d-12cd9af04474/Navigating-your-Healthcare-Final.pdf> | Charity  Review of published research | Help persons with OA to navigate healthcare system, understand treatment options and self-management strategies  General OA | 23 articles that describes patient empowerment, preparation to get most out of health care appointment, treatment team, OA medications and health-care system in Canada (funding health care and self-advocacy)  Self-management strategies like good nutrition, physical activities, heat and cold therapy and medication and mindfulness  Links to additional web resources  Thirteen resources: (1) Arthritis goal tracker (2) Health-care appointment checklist (3) Symptom tracker (4) Symptom checker (5) Post appointment record (6) Adverse reaction record form (7) Access to primary care (8) surgery (9) Advanced Practice Arthritis Therapist (10) Medication reference guide and (11) Provincial, Territorial and Indigenous Health Plans (12) Advocacy (13) Who’s who and who does what Chart | Booklet  54 pages | Available online to read or download |
| A Guide To Living With Osteoarthritis  Arthritis Consumer Experts  2018[84]  <https://www.jointhealth.org/pdfs/JointHealth-insight-2018-September.pdf> | Patient advocacy  Review of published research and expert advice from physiotherapists | Describe OA symptoms and self-management  General OA | Signs and symptoms of OA and self-management strategies such as exercise (aerobic activity and joint strengthening), weight loss, wearing suitable footwear, pain relief through heat and complementary therapies (e.g. Chiropractic, osteopathy etc.) with additional information on inflammatory arthritis and juvenile arthritis and checklist to help prepare for school year  Facts for happy joints that include move them before you use them, if your joints are tight, warm them and stretch them before using and move little bits often - even when you’re sore  Link to additional web resources  Three resources: (1) Facebook account Arthritis Consumer Experts (2) Twitter account @ACEJointHealth and (3) About Arthritis: Joint health | Booklet  7 pages | Available online to read or download |
| Patient Reference Guide  Health Quality Ontario  2018[85]  <https://www.hqontario.ca/Portals/0/documents/evidence/quality-standards/qs-osteoarthritis-patient-guide-en.pdf> | Government  Expert advice: Committee of healthcare professionals, persons living with OA, their family and caregivers | Help persons with OA to prepare for healthcare appointments  Knee, hip, or hand OA | Issues to discuss with health care providers and brief answers to following questions:   1. How Will I Know if I Have Osteoarthritis? 2. What is a Care Plan? 3. How Can I Manage My Osteoarthritis? 4. Who Will Be Involved in My Care? 5. Will I Need Pain Medication? 6. Will I Need Surgery?   Self-management strategies include education, exercise (therapeutic and physical activities), weight management and pain medication. | Booklet  10 Pages | Available online to read or download |
| Activity Diary  Arthritis Society  2018[86]  <https://arthritis.ca/AS/media/pdf/Support%20and%20Education/EN-activity-diary.pdf> | Charity  Not reported | Describe and track OA self management  General OA | Colour code to mark daily activities depending on amount of energy invested. For example:  Red for high energy  Yellow for low energy  Green for rest time  Blue for sleep  X sign for high fatigue  Activity chart to colour code activities conducted throughout the day from morning to night for whole week  List of questions to ask when recording activities   1. Are there times when I was exhausted? 2. Are these related to high-energy activities? 3. Are there long blocks of high-energy activity with no breaks? 4. Is my sleep disturbed? 5. Am I sleeping in the day? 6. Is there enough time for enjoyment and recovery? | Checklist  3 pages | Available online to read or download |
| Osteoarthritis  Alberta Health Services  2018[87]  <https://www.healthiertogether.ca/health-conditions/osteoarthritis/> | Government  Not reported | Describe OA risk and self-management  General OA | Introduction to OA  Risk factor include overweight, sports related injury, age- over 40, gender-women and hereditary links.  Ways to reduce OA risk include move more and maintain a healthy weight  Link to move more include   - Mild to moderate exercise like cardio or strengthening exercise - Introduction to Physical activity and their level - Physical activity requirement for adults 18-64 years and 65 years and older - Examples of accommodating physical activities into daily life - Ideas to make physical activity more fun such as outdoor activities, join sports, bike ride etc. - Tips to create successful change with setting a plan, budding up and self-reward - Solution to physical activity barriers - Tips before starting physical activities   Link to Maintaining healthy weight include   - Introduction to healthy weight - BMI Calculator - Lifestyle change myth and fact - Ideas to eat healthy include home cooked meals, build healthy plate, increase water intake, limit high fat and sugar, limit eating out, write what you eat and replace processed snacks - Incorporate Physical activity with example of moderate activity such as brisk walk, shooting hoops, daily chores and vigorous activities like jogging, cycling and skiing - Risk of lower level of sleep   Links to additional web-resources:  Four resources: (1) Fitting Physical activity into your day(2) Guidelines for adults 18-64 years (3) Guidelines for adults 65 years and older and (4) Physical activity counselling toolkit | Information organized by topic sub-headings | Multiple webpages |
| Healthcare Appointment Checklist  Arthritis Society Canada  2015[88]  <https://www.arthritis.ca/AS/media/pdf/Living%20Well/Appointment-Checklist-EN-v3.pdf> | Charity  Not reported | Help persons with OA to prepare for healthcare appointments  General OA | List of factors to consider or make notes of organized by a few weeks before, a few days before, the day before, and immediately after an appointment  Links to additional web-resources  Two tools: (1) symptom tracker and (2) Joint pain symptom checker | Checklist  1 page | Available online to read or download |
| NSAIDs & Osteoarthritis: Putting Risks Into Perspective  Rheum info  2013[89]  <https://rheuminfo.com/docs/living-with-arthritis/NSAIDs-and-OA-Putting-Risks-Into-Perspective-RheumInfo_EN.pdf> | Consortium  Expert advice: Rheumatologist | Describe OA medication  General OA | Side effects of NSAIDs include gastrointestinal (GI) effects and cardiovascular (CV) effects.  GI risk in perspective with example of GI incidents in in a city of 100,000 people in a year with everyone consuming NSAIDs and nobody consuming NSAIDs  CV risk in perspective with example of heart attacks incidents in a city of 100,000 people in a year with everyone consuming NSAIDs, nobody consuming NSAIDs , everyone is smoker and everyone has high blood pressure  List of symptoms that requires visiting a doctor   1. You are older than 75 2. You have had GI bleeding before 3. You are taking multiple NSAIDs (including low-dose ASA) 4. You are taking blood thinners like Coumadin (warfarin)   Tips for persons with OA taking NSAIDAs includes learn about the condition and medication and understand risk perspective | Infographic  1 page | Available online to read or download |
