# Supplementary material for: The Quality and Cultural Safety of Online Osteoarthritis Information for Affected Persons and Health Care Professionals: Content Analysis
Source: J Med Internet Res. 2024 Oct 18;26:e57698. doi: 10.2196/57698 (PMC11530738; doi:10.2196/57698)
Supplement: Multimedia Appendix 3 [file jmir_v26i1e57698_app3.docx]

Multimedia Appendix 3. Characteristics of included educational materials for health care professionals

| Material | | | Characteristics | | |
| --- | --- | --- | --- | --- | --- |
| Title Developer  Year published  [Reference]  Web address | Developer type  Approach | Objective  Type of OA | Content | Format  Pages (n) or Duration (min) | Delivery |
| Conservative OA Treatments – Examples for Providers  Alberta Bone &Joint Health Institute  2022 [90]  <https://toolkit.albertaboneandjoint.com/osteoarthritis/wp-content/uploads/7_Tables-of-Examples-for-the-Treatments-Toolbox-4.pdf> | Charity  Expert advice: conservative OA clinical committee | Describe OA treatment (pharmacological and non-pharmacological)  General OA | Conservative OA treatment categorized into core treatment and adjunct treatment. Core treatment includes education, exercise and physical activity and weight management  Adjunct treatment includes non-pharmacological and pharmacological treatment  Non-pharmacological treatments includes walking support, environment support, cognitive behaviour therapy and interdisciplinary self-management programs.  walking supports includes   1. Assistive devices like cane, crutches etc. 2. Braces like knee brace, hip brace etc. 3. Footwear and orthotics like custom or commercial foot orthotics, specialized footwear etc.   Environmental Supports includes   1. Assistive technologies like long shoehorn,sockaids etc. 2. Home adaptation like raised toilet seats, bath seat etc.   List of non-prescription treatment and prescription treatment such as oral and topical NSAIDs  Injectable medication examples like corticosteroid, hyaluronic acid and blood derived therapies | Infographic  2 pages | Available online to read or download |
| Knee'd: What to tell patient about knee injections for osteoarthritis  Canadian Healthcare Network  2022[91]  <https://www.canadianhealthcarenetwork.ca/kneed-what-tell-patients-about-knee-injections-osteoarthritis> | Consortium  Expert advice: Committee of orthopaedic surgeon and Rheumatologist and review of published research | Describe OA treatment (Pharmacological)  Knee OA | Common injections (corticosteroid) used for OA treatment, their benefit and concerns.  Alternative injections (hyaluronic acid, Platelet rich plasma injection) ,their benefit and evidence of their use | Text | Single web page |
| PEER simplified decision aid: osteoarthritis treatment options in primary care  College of Family Physicians of Canada  2020[92]  <https://www.cfp.ca/content/cfp/66/3/191.full.pdf> | Professional Society  Review of published research | To help clinicians discuss OA treatment options (pharmacological and non-pharmacological) with persons with OA  General OA | Estimated effectiveness of OA treatment options that include exercise and medicines such as intra-articular corticosteroids, duloxetine, NSAIDs, opioids, glucosamine, chondroitin, viscosupplementation and acetaminophen  Classification of treatments (by benefits and harms), withdrawals due to adverse events, potential adverse events, basic prescribing tips, and costs.  List of additional web-resource  One Tool: (1) Pain calculator | Infographic  3 pages | Available online to read or download |
| Osteoarthritis tool  Arthritis Alliance of Canada  College of Family Physicians of Canada  Centre for Effective Practice  2017 [93]  <https://jointhealth.org/pdfs/OATookKit_En.pdf> | Consortium (academic, charity, professional society)  Expert advice: Committee of clinical and system experts and review of published research | Algorithm to help clinicians identify symptoms, diagnose OA and offer treatment (pharmacological and non-pharmacological)  Knee, hip or hand OA | Three Section to record history, physical examination and diagnosis  History section includes questions related to pain pattern, duration and activities that persons with OA avoids and criteria for inflammatory consideration  Examination section for knee and hip includes space to record patient weight, height, vitals and findings from hip, knee examination for deformities, swelling, gait, sit to stand test, hip flexion, hip internal rotation, knee flexion Meniscus testing. Examination for hand section includes observation for swelling or deformities, grip and pinch test, squeeze test  Diagnosis section outlines the clinical assessment of hip, knee and hand and imaging consideration and referral to different type of care providers such as outpatient rehabilitation provider, sports and exercise medicine physician, pain specialist, rheumatologist and orthopaedic surgeon.  Management option includes both pharmacologic (For example Topical NSAIDs, Opioids etc.) and non-pharmacologic options (For example weight management, physical activity, assistive devices, thermal therapy etc.) | Infographic  8 pages | Available online to read or download |
