# Supplementary material for: The Quality and Cultural Safety of Online Osteoarthritis Information for Affected Persons and Health Care Professionals: Content Analysis
Source: J Med Internet Res. 2024 Oct 18;26:e57698. doi: 10.2196/57698 (PMC11530738; doi:10.2196/57698)
Supplement: Multimedia Appendix 4 [file jmir_v26i1e57698_app4.docx]

Multimedia Appendix 4. Quality of materials for persons with osteoarthritis evaluated using DISCERN* [34]

| Title Developer Year published[Ref] | Q1  Aims clear | Q2  Aims achieved | Q3  Relevant | Q4  Sources evident | Q5  Sources dated | Q6  Balanced | Q7  Further sources | Q8  Identifies uncertainties | Q9  Treatment options | Q10  Treatment benefits | Q11  Treatment risks | Q12  Outcomes without treatment | Q13  Treatment impact on quality of life | Q14  Treatment options compared | Q15  Supports shared decisions | Score  Items fully met/eligible items*100 | Quality** |
| --- | --- | --- | --- | --- | --- | --- | --- | --- | --- | --- | --- | --- | --- | --- | --- | --- | --- |
| How Can An OT Help People With Arthritis    Canadian Arthritis Patients Alliance  2023 [47] | Y | Y | Y | N | P | P | N | P | P | Y | N | N | P | Y | Y | 6/15=40.0% | Low |
| Osteoarthritis    Arthritis Society Canada  2023 [48] | Y | Y | Y | P | P | P | P | Y | N/A | N/A | N/A | N/A | N/A | Y | P | 5/10=50.0% | Moderate |
| What Is Osteoarthritis?  Arthritis Society Canada  2023 [49] | Y | P | P | N | P | P | Y | P | N | N | N | N | N | Y | Y | 4/15= 26.7% | Low |
| Complementary Therapies Guide For Arthritis  Arthritis Society Canada  2023[50] | Y | Y | Y | P | P | P | Y | Y | Y | Y | N | N | N | P | Y | 8/15=53.3% | Moderate |
| Appointment Discussion Guide  Arthritis Society Canada  2023[51] | Y | Y | Y | P | P | P | P | Y | N/A | N/A | N/A | N/A | N/A | N/A | Y | 5/9=55.5% | Moderate |
| Arthritis Symptom Checker  Arthritis Society Canada    2023[52] | Y | Y | Y | P | P | Y | P | Y | N/A | N/A | N/A | N/A | N/A | N/A | Y | 6/9= 66.7% | Moderate |
| Daily Symptom Tracker  Arthritis Society Canada  2023[53] | Y | Y | Y | P | P | P | N | N/A | N/A | N/A | N/A | N/A | Y | N/A | Y | 5/9= 55.5% | Moderate |
| Arthritis Risk Factor Assessment  Arthritis Society Canada  2023[54] | Y | Y | Y | N | N | Y | P | Y | N/A | N/A | N/A | N/A | N/A | N/A | Y | 6/9= 66.7% | Moderate |
| Joint Pain Symptom Checker  Arthritis Society Canada    2023[55] | Y | Y | Y | P | P | Y | Y | N/A | N/A | N/A | N/A | N/A | N/A | N/A | Y | 6/8=75.0% | High |
| Medication Reference Guide  Arthritis Society Canada  2023[56] | Y | Y | Y | P | P | Y | P | Y | Y | N | Y | N | N | P | Y | 8/15= 53.3% | Moderate |
| Low Load Activities For Osteoarthritis  Arthritis Society Canada  2023[57] | P | Y | Y | P | P | P | P | Y | P | Y | Y | N | Y | Y | Y | 8/15= 53.3% | Moderate |
| Modifying Activities For Osteoarthritis  Arthritis Society Canada  2023[58] | P | Y | Y | P | P | P | N | Y | N/A | N/A | N/A | N/A | N/A | N/A | N/A | 3/8= 37.5% | Low |
| Arthritis Screening Exam  Arthritis Consumer expert and Arthritis research Canada  2023[59] | Y | Y | Y | P | P | Y | Y | Y | N/A | N/A | N/A | N/A | N/A | N/A | Y | 7/9=77.8% | High |
| Tips On How To Manage Daily Cooking Tasks and Live Well With Arthritis  Canadian Arthritis Patient Alliance  2023[60] | Y | Y | Y | Y | P | P | P | P | P | P | N | N | N | Y | Y | 6/15=40.0% | Low |
| Tai Chi To Help Arthritis  Arthritis Society Canada  2022[61] | Y | Y | Y | P | P | P | P | N | Y | Y | N | P | Y | P | N | 6/15=40.0% | Low |
| Osteoarthritis  GLA: D Canada  2022[62] | Y | Y | Y | N | P | P | P | P | P | Y | P | Y | Y | Y | Y | 8/15=53.3% | Moderate |
| Osteoarthritis Symptoms And Diagnosis  Arthritis Society Canada  2021[63] | Y | Y | Y | P | P | P | Y | N/A | N/A | N/A | N/A | N/A | N/A | N/A | Y | 5/8=62.5% | Moderate |
| Osteoarthritis Self-Management  Arthritis Society Canada  2021[64] | Y | Y | Y | P | P | P | Y | Y | Y | Y | P | Y | Y | Y | Y | 11/15=73.3% | High |
| Osteoarthritis Treatment  Arthritis Society Canada  2021[65] | Y | Y | Y | P | P | P | P | Y | Y | Y | P | P | Y | Y | Y | 9/15=60.0% | Moderate |
| Updates In Osteoarthritis Treatment And Care With Dr. Tom Appleton  Joint health: Arthritis Consumer Experts  2021[66] | Y | Y | Y | P | P | P | Y | p | P | P | P | P | P | Y | Y | 6/15=40.0% | Low |
| Living Your Best Life with Osteoarthritis  Alberta Health Services,  Alberta Bone and Joint Health Institute  2020[67] | Y | Y | Y | P | P | P | Y | Y | P | Y | P | N | Y | Y | Y | 9/15= 60.0% | Moderate |
| Introduction To Exercise For Osteoarthritis  Arthritis Society Canada  2020[68] | Y | Y | Y | P | P | P | P | P | Y | Y | P | Y | Y | Y | Y | 9/15=60.0% | Moderate |
| 20-Minute Warm-Up For The Joints  Arthritis Society Canada  2020[69] | Y | Y | Y | N | P | P | N | Y | P | P | N | N | N | Y | Y | 6/15=40.0% | Low |
| OA: Patient Journey  Arthritis Society Canada  2020[70] | Y | Y | Y | N | N | Y | Y | Y | P | P | P | P | Y | Y | Y | 9/15= 60.0% | Moderate |
| Assistive Devices Resource  Arthritis Society Canada  2020[71] | Y | Y | Y | P | P | P | N | P | Y | Y | N | N | P | Y | P | 6/15=40.0% | Low |
| Drug Free Pain Management Tool  Arthritis Society Canada  2020[72] | Y | Y | Y | P | P | P | Y | Y | Y | Y | P | P | Y | Y | Y | 10/15=66.7% | Moderate |
| Medical Cannabis And Arthritis  Arthritis Society Canada  2019[73] | Y | Y | Y | Y | P | Y | Y | Y | Y | Y | Y | N | Y | N/A | Y | 12/14= 85.7% | High |
| Talk To Your Doctor about Joint Pain  Arthritis Alliance of Canada  2019[74] | Y | Y | Y | N | P | P | Y | Y | N/A | N/A | N/A | N/A | N/A | N/A | Y | 6/9= 66.7% | Moderate |
| Exercises For Arthritis  University of Alberta  2019[75] | Y | Y | Y | Y | Y | Y | P | P | Y | P | P | N | P | Y | Y | 9/15=60.0% | Moderate |
| Managing Chronic Pain Online Learning Module  Arthritis Society Canada  2018[76] | Y | Y | Y | Y | Y | Y | Y | Y | Y | Y | P | P | P | Y | Y | 12/15=80.0% | High |
| Overcoming Fatigue Online Learning Module  Arthritis Society Canada  2018[77] | Y | Y | Y | Y | Y | Y | Y | Y | Y | Y | P | P | Y | Y | Y | 13/15=86.7% | High |
| Daily Living Online Learning Module  Arthritis Society Canada  2018[78] | Y | Y | Y | Y | Y | Y | Y | Y | Y | Y | P | P | Y | Y | Y | 13/15=86.7% | High |
| Mental Health & Well-being Online Learning Module  Arthritis Society Canada  2018[79] | Y | Y | Y | Y | Y | Y | Y | Y | P | P | N | Y | Y | Y | Y | 12/15=80.0% | High |
| Arthritis And Work Online Learning Module  Arthritis Society Canada  2018[80] | Y | Y | Y | Y | Y | Y | Y | Y | P | Y | N | P | Y | Y | Y | 12/15=80.0% | High |
| Staying Active Online Learning Module  Arthritis Society Canada  2018[81] | Y | Y | Y | Y | Y | Y | Y | Y | Y | P | N | Y | Y | Y | Y | 13/15= 86.7% | High |
| Eating Well Online Learning Module  Arthritis Society Canada  2018[82] | Y | Y | Y | Y | P | Y | Y | Y | P | Y | Y | N | Y | Y | Y | 12/15= 80.0% | High |
| Navigating Your Healthcare online Learning Module  Arthritis Society Canada  2018[83] | Y | Y | Y | Y | P | Y | Y | Y | Y | Y | Y | P | N/A | Y | Y | 12/14= 85.7% | High |
| A Guide To Living With Osteoarthritis  Arthritis Consumer Experts  2018[84] | N | N/A | P | P | P | Y | Y | Y | Y | Y | P | N | Y | Y | Y | 8/14=57.1% | Moderate |
| Patient Reference Guide  Health quality Ontario  2018[85] | Y | Y | Y | N | N | P | P | Y | Y | Y | Y | N | Y | Y | Y | 10/15= 66.7% | Moderate |
| Activity Diary  Arthritis Society  2018[86] | Y | Y | Y | N | P | P | N | N/A | N/A | N/A | N/A | N/A | N/A | N/A | P | 3/8=37.5% | Low |
| Osteoarthritis  Alberta Health Services  2018[87] | Y | Y | Y | Y | Y | Y | Y | Y | Y | Y | N | P | Y | Y | Y | 13/15=86.7% | High |
| Healthcare Appointment Checklist  Arthritis Society Canada  2015[88] | Y | Y | Y | P | P | P | N | N/A | N/A | N/A | N/A | N/A | N/A | N/A | Y | 4/8= 50.0% | Moderate |
| NSAIDs & Osteoarthritis: Putting Risks Into Perspective  Rheum info  2013[89] | Y | Y | Y | N | P | P | P | P | P | Y | Y | N | N | N | Y | 6/15=40.0% | Low |

*DISCERN scale: No 1; Partial 2-4; Yes 5

**Quality: 70%+ high quality, 50% to 69% moderate quality, <50% low quality
