# Supplementary material for: The Quality and Cultural Safety of Online Osteoarthritis Information for Affected Persons and Health Care Professionals: Content Analysis
Source: J Med Internet Res. 2024 Oct 18;26:e57698. doi: 10.2196/57698 (PMC11530738; doi:10.2196/57698)
Supplement: Multimedia Appendix 5 [file jmir_v26i1e57698_app5.docx]

Multimedia Appendix 5. Quality of materials for healthcare professionals

| Title Developer  Year published  [Reference] | Q1  Self directed | Q2  Needs based | Q3  Case based | Q4  Evidence based | Q5  Interactive | Q6  Support for practice | Q7  Credible | Q8  Format choice | Score  Items met /eligible items*100 | Quality* |
| --- | --- | --- | --- | --- | --- | --- | --- | --- | --- | --- |
| Conservative OA Treatments – Examples for Providers  Alberta Bone &Joint Health Institute  2022 [90] | Y | Y | N | N | N | Y | N | Y | 4/8=50.0% | Moderate |
| Knee'd: What to tell patients about knee injections for osteoarthritis  Canadian Healthcare Network  2022[91] | Y | Y | N | N | N | N | N | N | 2/8= 25.0% | Low |
| PEER simplified decision aid: osteoarthritis treatment options in primary care  College of Family Physicians of Canada  2020[92] | Y | Y | N | Y | N | Y | Y | Y | 6/8=75.0% | High |
| Osteoarthritis tool  Arthritis Alliance of Canada  College of Family Physicians of Canada  Centre for Effective Practice  2017 [93] | Y | Y | N | Y | Y | Y | Y | Y | 7/8=87.5% | High |

*Quality: 70%+ high quality, 50% to 69% moderate quality, <50% low quality
