# Supplementary material for: The Quality and Cultural Safety of Online Osteoarthritis Information for Affected Persons and Health Care Professionals: Content Analysis
Source: J Med Internet Res. 2024 Oct 18;26:e57698. doi: 10.2196/57698 (PMC11530738; doi:10.2196/57698)
Supplement: Multimedia Appendix 6 [file jmir_v26i1e57698_app6.docx]

Multimedia Appendix 6. Cultural safety of materials for persons with osteoarthritis*

| Title Developer Year published  [Reference] | Language and communication | | | Accommodation of cultural beliefs, values and norms | Solutions to overcome barriers | Overall score | Cultural safety** |
| --- | --- | --- | --- | --- | --- | --- | --- |
|  | Plain language | Translated versions | Empathetic communication |  |  |  |  |
| How Can An OT Help People With Arthritis?  Canadian Arthritis Patient Alliance  2023[47] | Y | N | Y | N | N | 2/5=40.0% | Low |
| Osteoarthritis  Arthritis Society Canada  2023 [48] | Y | N | Y | Y | N | 3/5=60.0% | Moderate |
| What Is Osteoarthritis?  Arthritis Society Canada  2023 [49] | Y | N | Y | N | N | 2/5=40.0% | Low |
| Complementary Therapies Guide For Arthritis  Arthritis Society Canada  2023[50] | Y | N | Y | Y | Y | 4/5=80.0% | High |
| Appointment Discussion Guide  Arthritis Society Canada  2023[51] | Y | N | Y | N | Y | 3/5=60.0% | Moderate |
| Arthritis Symptom Checker  Arthritis Society Canada    2023[52] | Y | N | Y | N | N | 3/5=60.0% | Moderate |
| Daily Symptom Tracker    Arthritis Society Canada  2023[53] | Y | N | Y | N | Y | 3/5=60.0% | Moderate |
| Arthritis Risk Factor Assessment    Arthritis Society Canada    2023[54] | N | N | Y | N | Y | 2/5=40.0% | Low |
| Joint Pain Symptom Checker  Arthritis Society Canada  2023[55] | N | N | Y | N | N | 1/5=20.0% | Low |
| Medication Reference Guide  Arthritis Society Canada  2023[56] | N | N | Y | N | Y | 2/5=40.0% | Low |
| Low Load Activities For Osteoarthritis  Arthritis Society Canada  2023[57] | Y | N | Y | Y | Y | 4/5=80.0%% | High |
| Modifying Activities For Osteoarthritis  Arthritis Society Canada  2023[58] | Y | N | Y | Y | N | 3/5=60.0% | Moderate |
| Arthritis Screening Exam  Arthritis Consumer Expert and Arthritis Research Canada  2023[59] | N | N | Y | N | Y | 2/5=40.0% | Low |
| Tips On How To Manage Daily Cooking Tasks and Live Well With Arthritis  Canadian Arthritis Patient Alliance  2023[60] | Y | N | Y | Y | Y | 4/5=80.0%% | High |
| Tai Chi To Help Arthritis  Arthritis Society Canada  2022[61] | Y | N | Y | Y | Y | 4/5=80.0%% | High |
| Osteoarthritis  GLA: D Canada  2022[62] | N | N | Y | Y | Y | 3/5=60.0% | High |
| Osteoarthritis Symptoms And Diagnosis  Arthritis Society Canada  2021[63] | Y | N | Y | Y | N | 3/5=60.0% | Moderate |
| Osteoarthritis Self-Management  Arthritis Society Canada  2021[64] | N | N | Y | Y | Y | 3/5=60.0% | Moderate |
| Osteoarthritis Treatment  Arthritis Society Canada  2021[65] | N | N | Y | Y | N | 2/5=40.0% | Low |
| Updates In Osteoarthritis Treatment And Care With Dr. Tom Appleton  Joint health: Arthritis Consumer Experts  2021[66] | N | N | Y | Y | Y | 3/5=60.0% | Moderate |
| Living Your Best Life With Osteoarthritis  Alberta Health Services,  Alberta Bone and Joint Health Institute  2020[67] | Y | N | Y | Y | Y | 4/5=80.0% | High |
| Introduction To Exercise For Osteoarthritis  Arthritis Society Canada  2020[68] | Y | N | Y | N | Y | 3/5=60.0% | Moderate |
| 20-Minute Warm-Up For The Joints  Arthritis Society Canada  2020[69] | N | N | Y | N | Y | 2/5=40.0% | Low |
| OA: Patient Journey  Arthritis Society Canada  2020[70] | Y | N | Y | Y | Y | 4/5=80.0% | High |
| Assistive Devices Resource  Arthritis Society Canada  2020[71] | Y | N | Y | N | N | 2/5=40.0% | Low |
| Drug Free Pain Management Tool  Arthritis Society Canada  2020[72] | N | N | Y | N | Y | 2/5=40.0% | Low |
| Medical Cannabis And Arthritis  Arthritis Society Canada  2019[73] | N | N | Y | N | Y | 2/5=40.0% | Low |
| Talk To Your Doctor About Joint Pain  Arthritis Alliance of Canada  2019[74] | Y | N | Y | N | Y | 3/5=60.0% | Moderate |
| Exercises For Arthritis  University of Alberta  2019[75] | Y | N | Y | Y | Y | 4/5=80.0% | High |
| Managing Chronic Pain Online Learning Module  Arthritis Society Canada  2018[76] | N | N | Y | Y | Y | 3/5=60.0% | Moderate |
| Overcoming Fatigue Online Learning Module  Arthritis Society Canada  2018[77] | N | N | Y | Y | Y | 3/5=60.0% | Moderate |
| Daily Living Online Learning Module  Arthritis Society Canada  2018[78] | N | N | Y | Y | Y | 3/5=60.0% | Moderate |
| Mental Health & Well-being Online Learning Module  Arthritis Society Canada  2018[79] | N | N | Y | Y | Y | 3/5=60.0% | Moderate |
| Arthritis And Work Online Learning Module  Arthritis Society Canada  2018[80] | N | N | Y | Y | Y | 3/5=60.0% | Moderate |
| Staying Active Online Learning Module  Arthritis Society Canada  2018[81] | Y | N | Y | Y | Y | 4/5=80.0% | High |
| Eating Well Online Learning Module  Arthritis Society Canada  2018[82] | Y | N | Y | N | Y | 3/5=60.0% | Moderate |
| Navigating Your Healthcare online Learning Module  Arthritis Society Canada  2018[83] | Y | N | Y | N | Y | 3/5=60.0% | Moderate |
| A Guide To Living With Osteoarthritis  Arthritis Consumer Experts  2018[84] | Y | N | Y | N | Y | 3/5=60.0% | Moderate |
| Patient Reference Guide  Health quality Ontario  2018[85] | Y | N | Y | Y | Y | 4/5=80.0% | High |
| Activity Diary  Arthritis Society  2018[86] | Y | N | Y | N | Y | 3/5=60.0% | Moderate |
| Osteoarthritis  Alberta Health Services  2018[87] | Y | N | Y | Y | Y | 4/5=80.0% | High |
| Healthcare Appointment Checklist  Arthritis Society Canada  2015[88] | Y | N | Y | N | Y | 3/5=60.0% | Moderate |
| NSAIDs & Osteoarthritis: Putting Risks Into Perspective  Rheum info  2013[89] | Y | N | Y | N | Y | 3/5=60.0% | Moderate |

*Scale: N (material does not fulfil criteria) or Y (material fulfills criteria)

** Cultural safety: 70%+ high cultural safety, 50% to 69% moderate cultural safety, <50% low cultural safety
