# Supplementary material for: The Quality and Cultural Safety of Online Osteoarthritis Information for Affected Persons and Health Care Professionals: Content Analysis
Source: J Med Internet Res. 2024 Oct 18;26:e57698. doi: 10.2196/57698 (PMC11530738; doi:10.2196/57698)
Supplement: Multimedia Appendix 7 [file jmir_v26i1e57698_app7.docx]

Multimedia Appendix 7. Cultural safety of osteoarthritis materials for healthcare professionals*

| Title Developer  Year published  [Reference] | Disparities | Cultural Awareness | Strategies to address cultural barriers | Total Score | Cultural safety** |
| --- | --- | --- | --- | --- | --- |
| Conservative OA Treatments – Examples for Providers  Alberta Bone &Joint Health Institute  2022 [90] | N | N | N | 0/3=0.0% | Low |
| Knee'd: What to tell patient about knee injections for osteoarthritis  Canadian Healthcare Network  2022[91] | N | N | N | 0/3=0.0% | Low |
| PEER simplified decision aid: osteoarthritis treatment options in primary care  College of Family Physicians of Canada  2020[92] | N | N | N | 0/3=0.0% | Low |
| Osteoarthritis tool  Arthritis Alliance of Canada  College of Family Physicians of Canada  Centre for Effective Practice  2017 [93] | N | N | Y | 1/3=33.3% | Low |

*Scale: N (material does not fulfil criteria) or Y (material fulfils criteria)

** Cultural safety: 70%+ high cultural safety, 50% to 69% moderate cultural safety, <50% low cultural safety
